# Population Pharmacokinetics of Intraventricular Vancomycin in Neonatal Ventriculitis, A Preterm Pilot Study

**DOI:** 10.1016/j.ejps.2020.105643

**Published:** 2021-03-01

**Authors:** Jaya Madhura Parasuraman, Frank Kloprogge, Joseph Frank Standing, Mahableshwar Albur, Axel Heep

**Affiliations:** a Neonatal Intensive Care Unit, Southmead Hospital, Southmead Road, Bristol, United Kingdom, BS10 5NB; b Infection, Inflammation and Rheumatology Section, Institute of Child Health, University College London, 30 Guilford Street, Holborn, London, United Kingdom, WC1N 1EH; c Institute for Global Health, University College London, 30 Guilford Street, Holborn, London, United Kingdom, WC1N 1EH; d Department of Medical Microbiology, Southmead Hospital, Southmead Road, Bristol, United Kingdom, BS10 5NB; e Neonatal Neurology Group, School of Clinical Sciences, University of Bristol, Bristol, United Kingdom, BS8 1TH

**Keywords:** intraventricular vancomycin, neonatal ventriculitis, preterm pharmacokinetics, NONMEM modelling

## Abstract

**Aim:**

Intraventricular vancomycin is an effective treatment for neonatal ventriculitis, as the cerebrospinal fluid (CSF) vancomycin levels reach adequate concentrations to achieve microbiological cure. There is no robust data on intraventricular vancomycin pharmacokinetics in the preterm population. This pilot population pharmacokinetic modelling study examines the pharmacokinetic behaviour of intraventricular vancomycin in the preterm population of < 28 weeks gestation, to inform the feasibility of future prospective studies.

**Methods:**

The study comprised 8 preterm infants with neonatal ventriculitis (median gestation age 25.3 weeks; range 23.9 - 27.7). Population pharmacokinetics (non-linear mixed effects modelling) were described with one- and two-compartment models to fit plasma concentrations of vancomycin. A CSF compartment was added to the plasma modelling and mass transfer examined. Three covariates (serum creatinine, ventricular index (VI) and CSF protein) were tested on the final model. Area under the curve (AUC) and average CSF concentration (C average) predictions were generated from the final model and compared with time to microbiological cure.

**Results:**

A one-compartment model provided the best fit to the data. There was no appreciable transfer between plasma and CSF. None of the covariates provided a significant reduction in the objective function value (OFV). Generally, time to sterilisation with higher CSF AUC (0-24) and C average tends to be shorter, however this should be interpreted with caution as data is erratic.

**Conclusion:**

This pilot population pharmacokinetic analysis provides important information to warrant changes in the management of intraventricular vancomycin treatment in the preterm population, such as the current use of VI as a dosing parameter. Further study with a larger data pool is necessary to investigate the influence of VI on CSF vancomycin and ascertain dosing strategies.

## Highlights

• Pharmacokinetics modelling of intraventricular vancomycin in a preterm pilot study.
• Intraventricular vancomycin follows a one compartment model in neonatal ventriculitis treatment.
• Ventricular Index, a dosing parameter, does not influence cerebrospinal fluid vancomycin levels.

## Graphical abstract

**Figure:**
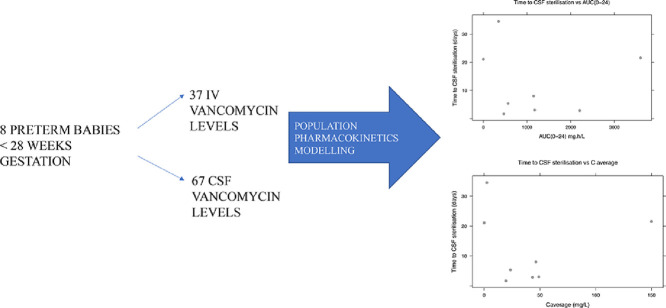

## 1 Introduction

Intraventricular vancomycin is increasingly used to treat ventriculitis, especially in the neonatal population, either as the first line or concomitantly with intravenous (IV) vancomycin (Li et al., 2017; Matsunaga et al., 2015). This condition affects up to 6% of preterm babies, usually with preceding history of intraventricular haemorrhage (IVH) and post haemorrhagic hydrocephalus as a consequence of IVH (Brouwer et al., 2015). They hence require external cerebrospinal fluid (CSF) management through a ventricular access device. The frequency of taps and factors linked to prematurity such as fragile skin places them at a higher risk for ventriculitis, although the ventriculitis rate in the adult population is similar at approximately 6-8% (Sam et al., 2018; Park et al., 2004).

The neurodevelopmental impairment burden in neonatal ventriculitis is yet unquantified in the literature, but it is known that having a shunt alone in post haemorrhagic hydrocephalus increases the risk of neurodevelopmental impairment and outcomes of growth at 18-22 months (Adams-Chapman et al., 2008). Although data is scarce on neurodevelopmental impairment outcomes in neonatal ventriculitis, a recent meta analyses demonstrated that 18% of infantile GBS meningitis survivors suffered moderate to severe neurodevelopmental impairment (Kohli-Lynch et al., 2017). This can be extrapolated to the ventriculitis population. The added risk of ventriculitis in this group of already vulnerable infants necessitates a better evidence base in order to influence the management of this condition.

Although vancomycin has been used widely in the neonatal population, there is still limited knowledge on its pharmacokinetics, regardless of the route of administration (Jacqz-Aigrain et al., 2013). IV vancomycin is the antibiotic of choice in late onset neonatal sepsis, when staphylococci infections are suspected and particularly in the presence of indwelling catheters. IV vancomycin pharmacokinetics exhibits large interindividual variability (Jacqz-Aigrain et al., 2013). Amelie et al reviewed 15 population pharmacokinetic models of intravenous vancomycin that included neonatal and paediatric population and identified that one-compartment model best described vancomycin in this population, and this is consistent with earlier work (Marsot et al., 2012; Seay et al., 1994). Age, body weight and creatinine clearance were found to be the covariates associated with dose optimization (Marsot et al., 2012).

In a review of vancomycin use in neonates over three decades by De Hoog et al in 2004, volume of distribution of intravenous vancomycin in steady state in neonates was found to range between 0.38 and 0.97 L/kg and the clearance ranged between 0.04 to 0.09 L/hour/kg (de Hoog et al., 2004). Furthermore, a large range of peak and trough levels of vancomycin were found to be effective against Gram positive infections in the neonatal population, and there is lack of literature to support the widely used therapeutic serum trough concentrations of 5-10mg/L (de Hoog et al., 2004). The authors of this review also found no data on the pharmacokinetics of intraventricular vancomycin (de Hoog et al., 2004). British National Formulary for Children suggests a serum trough level of 10-20mg/L under normal circumstances and recommends a trough concentration of 15-20 mg/L to treat organisms with MIC of 1mg/L or higher (British National Formulary for Children, 2018). Furthermore, this formulary advises a trough concentration of < 10mg/L for intraventricular vancomycin, although this target is not specific to the neonatal population (British National Formulary for Children, 2018).

Staphylococci species are the predominant causative organisms in neonatal ventriculitis (Adams-Chapman et al., 2008; Parasuraman et al., 2018). A previous study by Nagl et al showed that the microbiocidal effects of vancomycin against *Staphylococcus aureus* and *Staphylococcal epidermidis* was noted to be maximum at 5-10 mg/L, when the minimum inhibitory concentration (MIC) was 2mg/L, in adult CSF samples (Nagl et al., 1999). However, IV vancomycin is only able to achieve CSF vancomycin concentrations of 1.1-6mg/L, across the adult and the neonatal population (Li et al., 2015; Beach et al., 2017; Reiter and Doron, 1996). Intraventricular vancomycin use in neonatal ventriculitis, although has limited data in the literature, has been described through case reports and retrospective clinical data reviews (Matsunaga et al., 2015; Pau et al., 1986). A recent review by Matsunaga et al suggested that a dose of 5mg of intraventricular vancomycin is sufficient as a starting dose to treat neonatal ventriculitis, producing CSF vancomycin levels of 16.5mg/L at 72 hours post administration (Matsunaga et al., 2015).

We have previously examined the relationship of 4 different intraventricular vancomycin doses, between 3mg to 15mg, and the CSF vancomycin concentrations, in a small cohort of preterm infants of < 28 weeks gestation (Parasuraman et al., 2018). In the four different dose models, the CSF vancomycin levels were adequately maintained at a level of at least ten times above the breakpoint of MIC (>10 mg/L) for coagulase-negative staphylococci (CoNS) (Parasuraman et al., 2018). There were also no confirmed complications, specifically renal effects and ototoxicity, seen with the CSF vancomycin level ranging from 2.5-230.7 mg/L (Parasuraman et al., 2018). In this study, we could not obtain additional CSF samples to study the intraventricular vancomycin pharmacokinetics in more details as the study design was of retrospective nature.

Using our previously published data, we have now applied a population pharmacokinetics approach with the aim to study the pharmacokinetic behaviour of intraventricular vancomycin in the preterm population, to inform the feasibility of future prospective studies.

## 2 Methods

### 2.1 Data Collection: Vancomycin (IV/Intraventricular vancomycin) dosing and sampling

A retrospective clinical case review of ventriculitis in preterm infants of < 28 weeks gestation was undertaken in Southmead Hospital Neonatal Intensive Care Unit (NICU), in Bristol, United Kingdom. The study period spans from 2009 to 2016. Over this 8-year study period, 8 preterm patients that were treated for ventriculitis, were identified. In this patient cohort, ventriculitis was defined as elevated CSF white cell count (WCC) >20/mm***^***^3^ or positive microbiological CSF culture, obtained through daily CSF drainage via ventricular access device (Ommaya reservoir).

Patient medical records both written and electronic were utilised to collect demographical and pharmacokinetic data on IV vancomycin as well as intraventricular vancomycin. For every patient, the following information was collected:

CSF WCC, microbiological CSF culture results, CSF protein levels, IV vancomycin and intraventricular vancomycin doses administered, all available CSF vancomycin levels and serum vancomycin levels, dates and times of IV vancomycin doses, intraventricular vancomycin doses, serum and CSF vancomycin levels.

The intraventricular vancomycin treatment doses for the preterm infants were individualised, as per the recommendations by the Medical Microbiologists and in discussion with the attending Consultant Neonatologist.

Four starting doses were used in the study's preterm cohort of 3mg, 5mg, 10mg and 15mg.

The infants had cranial ultrasound (USS) studies 2-3 times per week, using a Philips HD5 USS machine, through an 8.5 MHz sector transducer. Specific measurement of ventricular index (VI), which is defined as the distance between the falx and the lateral wall of the anterior horn in the coronal plane, was undertaken (Brouwer et al., 2012). The VI measurements were plotted on a Levene's centile chart, and ventricular size classed as per the following: no ventricular dilatation (up to 97th centile), mild ventricular dilatation (> 97th centile and up to 97th centile +2 mm), moderate ventricular dilatation (> 97th centile +2 mm and up to 97th centile +4 mm) and severe ventricular dilatation (> 97th centile + 4 mm) (Levene, 1981).

Intraventricular vancomycin was given when the ventricular access device was accessed for the routine drainage. Intraventricular vancomycin concentration used was 10 mg/mL, in a sterile preparation, and was given as a slow bolus over 2 minutes. The intraventricular vancomycin was given at the end of the CSF drainage. The ventricular access device was flushed with 1ml of sterile NaCl 0.9% following instillation of the intraventricular vancomycin.

CSF samples for vancomycin levels were taken when the ventricular access device was accessed for routine CSF drainage, as needed for clinical management of hydrocephalus, for the individual baby. Due to this routine practice, the CSF vancomycin levels were taken at random time points. Repeat intraventricular vancomycin dose was administered if the level dropped under the threshold of 10mg/L. The target CSF vancomycin concentration was at least ten times above the breakpoint of MIC for CoNS. This was chosen as these were the principal infecting organisms in the defined neonatal cohort and the study hospital's MIC breakpoint for CoNS is 1 mg/L (EUCAST MIC breakpoint, 4 mg/L) (EUCAST, 2017).

The CSF vancomycin measurements were undertaken in the local microbiology laboratory, which is a national referral centre for antibiotic assays, through quantitative microsphere system (QMS® Thermo Scientific)-based immunoassay (Thermo Scientific. QMS ® Vancomycin (VANCO), 2015). The CSF vancomycin assay test performance is provided in Table 1 below (Assay verification-Indiko Plus assays Vancomycin, 2017):

**Table 1:** CSF vancomycin assay test performance.

Analyte	Accuracy (%)	Inter and Intra Assay Precision (%)	Specificity	Analytical Range (mg/L)	Lab bias (%)	Lower limit of Quantification (mg/L)
Vancomycin	Low = <15.0 Med/High = <10.0	Low = <15.0 Med/High = <10.0	No known interferences	2.0 - 100	Mean/median negative bias 2-3	2.0

Time to sterilisation was defined as length of time taken for CSF WCC to fall to < 20/mm***^***3 and simultaneously achieve sterile CSF.

6/8 infants received concomitant IV vancomycin, at a standard starting dose of 15 mg/kg. Then after, the interval varied with gestational age. Babies who were < 29 weeks postmenstrual age received the subsequent doses at 24 hourly intervals and those who were 29-35 weeks postmenstrual age received the following doses at 12 hourly intervals. Pre-dose serum vancomycin level is obtained one hour prior to the administration of the 3^rd^ IV dose, and before the 3^rd^ subsequent IV doses then after (Western Neonatal Prescribing Group, 2014).

### 2.2 Non-Linear Mixed Effects (NLME) Modelling

IV and intraventricular vancomycin pharmacokinetics (PK) were evaluated using the nonlinear mixed-effects modelling software NONMEM version 7.3 (ICON PLC, Ellicott City, MD). The first order conditional estimation method with the interaction option (FOCE-I) was used to estimate PK parameters and variability. R (version 3.0.2) and PsN (version 5.18.2) tools were used for pre- and postprocessing.

The PK base model was a one-compartment model with first order elimination. The one-compartment PK parameters were clearance (CL) and apparent volume of distribution (V). The inter-individual variability (IIV) and inter-occasion variability (IOV) were assumed to follow a log-normal distribution (Eq 1):

(1) $$p_{i}=p_{TV}e^{\left(\eta +\pi \right)},$$

where p_i_ is the model parameter for the i^th^ patient, p_TV_ is the typical value of the parameter in the population, η is the departure from the typical value for an individual (IIV) and π is the departure of the parameter from one occasion to another (IOV).The residual variability was initially described using a combined proportional and additive error model.

Covariate testing was performed on both plasma and CSF PK parameters. Covariates were selected for testing based on their biological plausibility of affecting response. Covariates were deemed to be significant according to the likelihood ratio test (-2 × log-likelihood), if a drop in the objective function value (OFV) is 3.84 or higher with one parameter and 5.99 or higher with 2 parameters (p < 0.05).

One- and two-compartment models were tested to fit plasma concentrations of vancomycin independently using the NONMEM library ADVAN subroutines, to define the basic structural model. All PK parameters were *a priori* scaled for size and age (Germovsek et al., 2017; Germovsek et al., 2016). Weight was standardised to 70 kg using allometric scaling and the allometric exponents 0.632 was tested to the basic structural model. This weight standardization enables comparison of parameters across the population with different demographics (Germovsek et al., 2017; Holford, 1996 May). Maturation function was added to the basic model, as from prior knowledge, this is known to improve the neonatal pharmacokinetic model on clearance (Germovsek et al., 2016). Covariate testing was done using serum creatinine on plasma clearance.

Using a one-compartment modelling approach, CSF compartment was added to the plasma modelling. The CSF model equation, with the incorporated plasma model is given below:

$$\begin{array}{c} \text{dA}(p)/\text{dt}=-cl_{p}/v_{p}\times A(p)\\ \text{dA}(\text{csf})/\text{dt}=-cl_{\text{csf}}/v_{\text{csf}}\times A(\text{csf}) \end{array}$$

Where p is plasma, cl is clearance, v is volume, A is amount, csf is cerebrospinal fluid, dA(p)/dt is change in plasma concentrations with time and dA(csf)/dt is change in cerebrospinal fluid concentrations with time.

In the first instance, parallel dosing was used, and no transfer was assumed between plasma and CSF. Mass transfer in both directions (plasma to CSF; CSF to plasma) was tested using first-order rate constants. We tested 2 covariates of VI and CSF protein, on CSF clearance and volume and CSF clearance respectively.

For model evaluation, decrease in objective function value (OFV), plots of observed versus population predicted concentrations (DV-PRED), observed versus individual predicted concentrations (DV-IPRED), conditional weighted residuals versus time after dose (CWRES-TAD) were utilized. The final population model was evaluated through visual predictive check (VPC) (*n*=1000). Parameter uncertainty was obtained from the standard error of estimate through the covariance step.

Area under the curve (AUC) predictions were generated for CSF vancomycin from the final model. We explored the relationships between AUC 0-24 and average CSF concentration (C average; defined as AUC(0-t)/t; t = time) and time to sterilisation of CSF.

### 2.3 Ethics:

The protocol for the study was approved by National Health Service (NHS) England Health Research Authority (HRA). The committee did not require parental informed consent due to retrospective design and use of anonymised data collected by clinical staff who would normally have access to these records as part of their clinical work.

## 3 Results

Pharmacokinetic data from 8 preterm infants were available for NLME modelling. The median gestation age was 25.3 weeks (range 23.9 - 27.7) and the median postnatal age was 8.7 weeks (range 3.9-23.1). 41 plasma samples (from 6 out of 8 patients who received concomitant IV vancomycin) and 74 CSF samples were available for the pharmacokinetic modelling. One plasma sample was below lower limit of quantification and 1 plasma sample was >100mg/L, most likely attributed to sampling or analytical error. These 2 samples were excluded from the analysis. One patient received two courses of intraventricular vancomycin treatment. We have included only the first episode of treatment (without concomitant IV vancomycin) within our pharmacokinetic analysis for this patient, therefore a further 2 plasma and 7 CSF samples were excluded from the analysis. Patient demographics have been provided in Table 2. 37 plasma samples (from 5 out of 8 patients who received concomitant IV vancomycin in the final analysis) and 67 CSF samples were available for the final pharmacokinetic modelling.

**Table 2:** Summary of demographics and sampling characteristics.

n	8
Birth weight (kg) #	0.78 (0.517 - 1.13)
gestational age (weeks) #	25.3 (23.9 - 27.7)
postnatal age (weeks) #	8.7 (3.9-23.1)
postmenstrual age (weeks) #	34.4 (30.2 – 48.1)
females (%)	5 (62.5)
plasma sample per patient #+	5 (2-18)
CSF samples per patient#	7.5 (4-16)
plasma concentrations (mg/L) #	8.6 (2.2-26.6)
CSF concentrations (mg/L) #	24.9 (2.5-230.7)
plasma time after dose (h) #	5.59 (0-14)
CSF time after dose (h) #	7.39 (0.02-57.37)
creatinine (µmol/L) #	25 (16-47)
C-reactive protein (mg/L) #	7.5 (0.5-125)
CSF protein (g/L) #	3.97 (0.62-24.34)
ventricular index (mm) #	17.3 (14.1-34.6)

*Note:* CSF is cerebrospinal fluid, # is median (range), + indicates median for 5/8 patients who received concomitant IV vancomycin.

The structural model that provided the best fit to the data was a one-compartment model. A two-compartment model did not provide a significant drop in the OFV. When CSF data was added, the initial assumption was of no transfer between the CSF and plasma. The addition of a transfer rate constant either in both directions and just from plasma to CSF did not significantly improve the model fit. Hence, our pharmacokinetic model did not demonstrate an appreciable transfer between plasma and CSF.

**Fig. 1:**
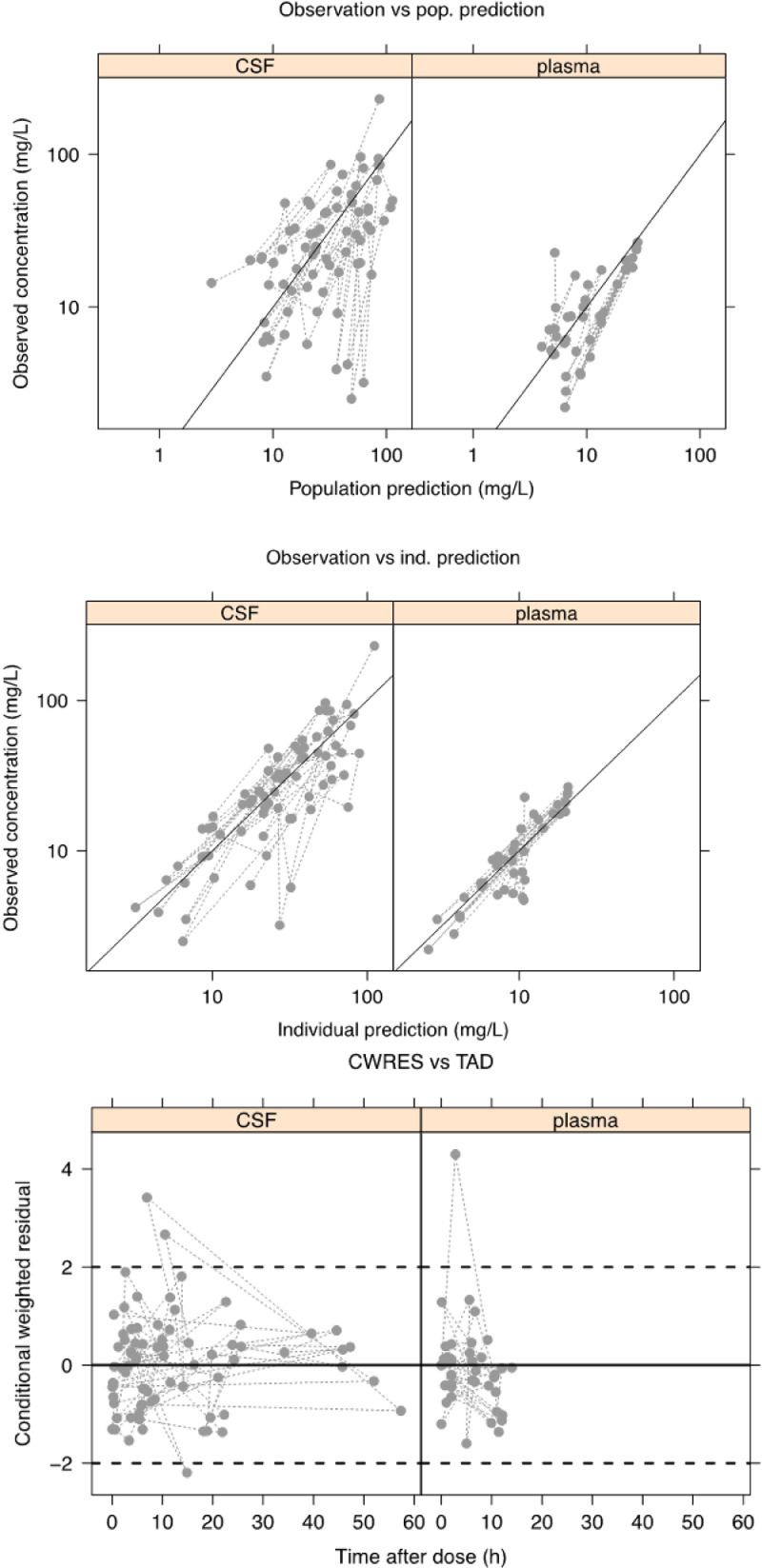
Basic goodness-of fit plots (DV-PRED, DV-IPRED, CWRES-TAD). Diagnostic plots showing observed versus population (top image) and individual (middle image) predicted cerebrospinal fluid (CSF) and plasma vancomycin concentration, and conditional weighted residuals against time after dose (bottom image).

**Fig. 2:**
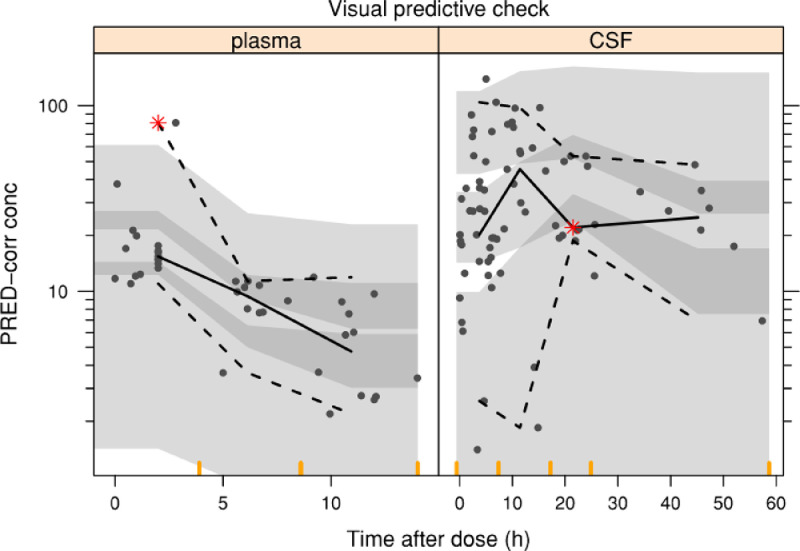
VPC Visual predictive check (n=1000) of vancomycin plasma (left) and cerebrospinal fluid (right) concentration versus time after dose; grey points are observations, black lines are the 2.5th, 50th and 97.5th percentiles of the observed data, and the shaded area is a non-parametric 95% confidence interval for the corresponding simulated concentrations. Red asterisks represent where the observed percentile falls outside the 95% confidence interval for the corresponding simulated concentrations.

We tested the covariates of serum creatinine, VI and CSF protein as described in section 2.2. The median values of VI and CSF protein from the patient cohort were used, at 17.3 mm and 3.97 g/L. None of the covariates provided a significant reduction in the OFV. The residual variability was initially described using a combined proportional and additive error model. We were unable to estimate a full covariance matrix due to numerical difficulties in the estimation process. In the final model IIV was estimated on CL, CL_csf and V_csf, and proportional error on plasma and CSF data. This strategy allowed a successful minimisation and covariance step and incorporated into the final model. There was no change in OFV from the initial parallel modelling of plasma and CSF and the final model (OFV=609.7). Model evaluation internally showed model predictions agreed with the observed concentrations, hence good fit to the data. No specific trend was observed in the residual plots for both plasma and CSF. These are represented by Fig. 1. Fig. 2 shows the results of prediction corrected VPC using 1000 replicates. The final estimates of model parameters are given in Table 3.

**Table 3:** Parameter estimates with uncertainty from the final model.

Parameter	Estimate (%RSE)	IIV % CV (%RSE)	Bootstrap median (95% CI)	Bootstrap IIV % CV (95%CI)
CL (L/h/70 kg)	9.29(5.2)	26(51.4)	9.5 (8.7 – 10.9)	25 (13 - 36)
V (L/70 kg)	54.4(11.5)	0 FIX	53.5 (47.6 – 119.8)	0 FIX
CLCSF (L/h)	0.002(12.9)	29(35.6)	0.002 (0.002 - 0.003)	27 (11 - 36)
VCSF (L)	0.109(23.8)	60(58)	0.11 (0.069 - 0.172)	59 (17 - 87)
sigmaprop plasma (% CV)	31.31(0.6)	-	30.1 (15.1 – 45.0)	-
sigmaprop CSF (% CV)	45.8(0.3)	-	44.8 (28.1 – 55.2)	-

*Note:* CL is clearance, V is volume of distribution, CSF is cerebrospinal fluid, CV is coefficient of variation $\left(100x\sqrt{\Omega }\right)$, IIV is inter individual variability, RSE is relative standard error $\left(\frac{\mathrm{standard}\;\mathrm{error}\;\mathrm{of}\;\mathrm{estimates}}{\mathrm{final}\;\mathrm{estimates}}\right)$, non-parametric bootstrap was derived from 1000 samples.

**Fig. 3:**
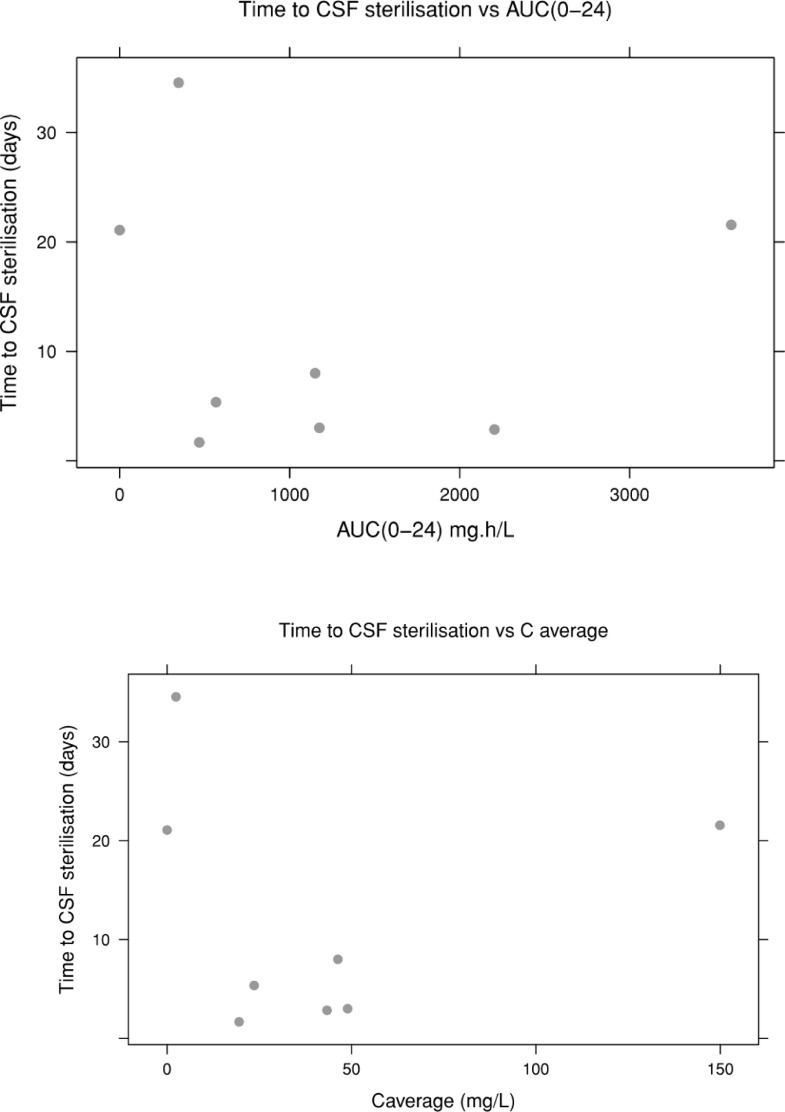
Time to CSF sterilisation versus AUC (0-24), top image, and C average, bottom image. Top image: y-axis denotes time to cerebrospinal fluid (CSF) sterilisation; x-axis denotes area under the curve (AUC 0-24). Bottom image: y-axis denotes time to cerebrospinal fluid (CSF) sterilisation; x-axis denotes C average (defined as AUC(0-t)/t; t = time).

The final model was used to generate CSF AUC predictions. The observed trends between AUC_0-24_ and C average suggest a shorter time to sterilisation with higher AUC (0-24) and C average, as represented in Fig. 3. However, it is acknowledged that within this limited dataset, the data is too erratic to interpret.

## 4 Discussion

A vancomycin population pharmacokinetic model was developed in preterm neonates receiving intraventricular and intravenous administration. Neonatal ventriculitis is a rare but significant complication in the described cohort of preterm babies, meaning that systematic study of large groups of patients is challenging. Robust pharmacokinetic data is vital to improve understanding of vancomycin administered by the intraventricular route to inform prescribing and treatment practice. In the preterm population, evidence is lacking on the currently used parameter of VI for intraventricular vancomycin dosing and the effects of important covariates on CSF vancomycin.

This pharmacokinetic modelling study demonstrates that a one-compartment model is the best fit for both the plasma and CSF data. Within the model and data, there was no appreciable transfer of vancomycin between plasma and CSF, in both directions. It is known from previous studies, such as by Reiter et al, the CSF vancomycin concentration is inadequate following intravenous vancomycin administration, between 2.2 and 5.6 mg/L (Reiter and Doron, 1996). A more recent work by Li et al highlighted that the disturbance of the blood brain barrier was important in transfer of IV vancomycin into the CSF, however they identified that the mean stable vancomycin concentration in the CSF was only 2 mg/L (Li et al., 2015). A systematic review by Beach et al in 2017 demonstrated that when IV vancomycin regimes between 2g-3.5g/day were used in patients with ventriculitis, CSF concentrations of 1.1-6 mg/L were achieved, which led to clinical cure (Beach et al., 2017). However, this study only reviewed 6 patients with ventriculitis, in an adult population, hence has several limitations (Beach et al., 2017). As stated in Section 1, Nagl et al demonstrated that the microbiocidal effects of vancomycin against *S.aureus* and *S.epidermidis* was noted to be maximum at 5-10 mg/L, when the MIC was 2mg/L, in adult CSF samples (Nagl et al., 1999). In our model, the undetectable transfer of vancomycin from plasma to CSF compartment is likely because of the relatively high level of CSF vancomycin concentration, due to the intraventricular vancomycin treatment, meaning that the relative contribution of IV vancomycin to CSF levels was small.

Pfausler et al examined the serum vancomycin concentration after intraventricular vancomycin administration of 10mg and found the level below measurable concentration (Pfausler et al., 2003). A further study by Li et al found that the vancomycin transferred from CSF into plasma was inconsequential due to the larger volume of plasma as compared to CSF (Li et al., 2017). The authors also established that the intraventricular administration is the main channel of vancomycin presence in the CSF (Li et al., 2017). As with our study, these findings point towards the one compartmental behaviour of vancomycin, especially when given intraventricularly, and hence may be the most effective route for treating intracranial infections. This is especially so in the preterm population, where failure of treatment may lead to adverse neurodevelopmental outcomes.

This study also explored the effects of covariates on the model and the CSF vancomycin concentrations. In particular, the effects of VI and CSF protein on CSF vancomycin volume (VI) and clearance (VI/CSF protein) were examined. The intraventricular vancomycin dosing in the patient cohort was guided by the ventricular size, through measurement of VI. This parameter is widely used in the neonatal population, however it is a surrogate marker for ventricular volume and not likely to accurately delineate volume of distribution. Currently, there is insufficient evidence on the use of ventricular size to determine the dose of intraventricular vancomycin, especially in the neonatal population, and the use of this parameter is based on local unit experience (Brown et al., 2000). In the adult population, Popa et al. demonstrated no significant relationship between CSF vancomycin concentration and ventricular size in patients who had received intraventricular vancomycin therapy (Popa et al., 2016).

The CSF protein was examined as a covariate in this study as 50-55% of vancomycin is protein bound (Schilling et al., 2011). As the CSF protein in a preterm baby is higher than that of a term infant, we hypothesised that this may have an effect on the unbound vancomycin in the CSF (Majumdar et al., 2013; De Cock et al., 2017). However, no significant association was found between VI, CSF protein and CSF vancomycin levels. This is an important finding in the preterm population, to review current intraventricular vancomycin prescribing parameters and practice.

This pharmacokinetic study of intraventricular vancomycin had a few limitations. Firstly, as the pharmacokinetic data was pooled from only 8 preterm patients, the results represent an association. We were also not able to perform intraventricular vancomycin dosing simulation, as doing so would not generate conclusive data due to the small patient cohort. Larger dataset is also needed to further explore and confirm the observed trend of shorter time to sterilisation with higher AUC (0-24) and C average. Furthermore, we did not examine the effects of CSF drainage on the CSF vancomycin level, as in our patient cohort the CSF drainage was fairly constant at 5-10ml/kg/day. There is some evidence to suggest that CSF drainage is associated with CSF vancomycin clearance (Li et al., 2018). This certainly makes physiological sense and has been behind the rationale for intraventricular vancomycin dosing recommendations in the adult population (Brown et al., 2000). From this pilot data, it is proposed that any future pharmacokinetic study on intraventricular vancomycin, in the preterm population, should firstly investigate the influence of VI on CSF vancomycin, as this is currently used as a dosing parameter and other covariates such as CSF drainage.

## 5 Conclusion

This pilot population pharmacokinetics study has demonstrated that a one-compartment model best described the pooled IV vancomycin and intraventricular vancomycin data from 8 preterm infants of < 28 weeks gestation. There was no appreciable transfer between plasma and CSF, hence intraventricular vancomycin is an effective route for treating ventriculitis. Covariates of VI and CSF protein did not demonstrate any influence on CSF vancomycin, and there was a trend of shorter time to sterilisation with higher AUC (0-24) and C average. Further study with larger data pool is necessary to investigate the influence of VI on CSF vancomycin in particular as the current dosing parameter and ascertain dosing strategies.

## Declarations of interest

None.

## Funding

This work was supported by the 10.13039/501100000395Royal College of Physicians (RCP) through Thomas Watts Eden Paediatric Fellowship (received by first author JMP), The Showering Fund from Southmead Hospital (grant number SF113-received by author MA) and United Kingdom Medical Research Council (MRC) Fellowships (grants P014534 and M008665-received by authors FK and JFS).

## Data Availability Statement

The data that support the findings of this study are available from the corresponding author upon reasonable request.

## CRediT authorship contribution statement

**Jaya Madhura Parasuraman:** Conceptualization, Methodology, Validation, Formal analysis, Investigation, Data curation, Writing - original draft, Writing - review & editing, Visualization, Project administration, Funding acquisition. **Frank Kloprogge:** Methodology, Software, Validation, Formal analysis, Investigation, Writing - review & editing, Visualization. **Joseph Frank Standing:** Conceptualization, Methodology, Software, Validation, Formal analysis, Investigation, Resources, Writing - review & editing, Visualization, Supervision. **Mahableshwar Albur:** Validation, Writing - review & editing. **Axel Heep:** Conceptualization, Methodology, Resources, Writing - review & editing, Supervision, Project administration, Funding acquisition.
